# Structure-based minimization of a high-mobility group protein B1 (HMGB1) B box peptide uncouples anti-NETotic activity and anti-biofilm function

**DOI:** 10.3389/fimmu.2026.1970789

**Published:** 2026-10-16

**Authors:** Kunal R. More, Jaime D. Rhodes, Lauren O. Bakaletz, Steven D. Goodman

**Affiliations:** 1 Center for Microbe and Immunity Research, Abigail Wexner Research Institute at Nationwide Children’s Hospital, Columbus, OH, United States; 2 Department of Pediatrics, College of Medicine, Ohio State University, Columbus, OH, United States

**Keywords:** biofilm inhibition, HMGB1, NET inhibition, NETs, peptide therapeutic, protein kinase C

## Abstract

**Introduction:**

We previously showed that human HMGB1 has anti-biofilm activity localized to a 97-amino-acid B-Box-derived peptide (mB Box-97), engineered with a C106S mutation to reduce HMGB1’s proinflammatory properties while retaining anti-biofilm function. This peptide also suppresses NETosis by inhibiting neutrophil protein kinase C (PKC) activity. Here, we tested whether the anti-biofilm and anti-NETotic activities of mB Box-97 could be separated into distinct shorter peptides by constructing truncated variants containing Helix 1 of the B-Box (with the C106S mutation), with or without the A Box–B Box linker region, and by introducing mutations at key DNA-binding residues.

**Methods:**

Anti-NETotic activity was assessed in human neutrophils, including peptide binding to PKC, effects on PKC activity, and downstream NADPH oxidase (NOX) activation, reactive oxygen species (ROS) generation, and NETosis. Biofilm disruption and bacterial growth effects were tested against pathogenic bacteria.

**Results:**

Peptides combining the linker with Helix 1 and C106S significantly inhibited NETosis while preserving anti-biofilm activity, via PKC binding/inhibition, reduced NOX activation, and lower ROS. Removing the linker preserved anti-biofilm activity but abolished anti-NETotic activity, yielding a 29-amino-acid anti-biofilm-only peptide. Conversely, adding DNA-binding mutations to the linker–Helix 1 peptide abolished anti-biofilm activity while retaining anti-NETotic function.

**Conclusions:**

Anti-NETotic and anti-biofilm activities of HMGB1-derived peptides can be structurally separated: the linker plus Helix 1 are required for anti-NETotic activity, while Helix 1 alone suffices for anti-biofilm activity. These compact 42- and 29-amino-acid peptides are readily synthesizable, promising candidates for biofilm- and NET-mediated inflammatory diseases, and further support PKC as a target for NETosis inhibition.

## 1 Introduction

Host-pathogen interactions are shaped by the push and pull between the host innate immune system and the ability of microorganisms to protect themselves. Neutrophils are a key component of the innate immune system, providing a primary line of defense against pathogens (1, 2). Through a process known as NETosis, polymorphonuclear leukocytes (PMNs) release decondensed chromatin decorated with antimicrobial proteins, forming structures called Neutrophil Extracellular Traps (NETs) (3, 4). These extracellular DNA structures can entrap free-living pathogens or encase microbial communities such as biofilms. In the case of free-living pathogens, the DNA scaffold locally concentrates bound antimicrobial proteins, thereby enhancing pathogen killing, the primary function of NETs (5).

NET formation can be triggered by diverse stimuli that typically converge on activation of the PKC-Raf/MEK/ERK pathway. One of the most extensively studied inducer is phorbol 12-myristate 13-acetate (PMA), which induces NET formation through PKC activation (6). Microbial infections can also trigger NETosis through PKC activation (1, 7, 8). Engagement of host Toll-like receptors (TLRs) by various microorganisms initiates nicotinamide adenine dinucleotide phosphate (NADPH) oxidase (NOX) dependent NETosis; for example, recognition of *Streptococcus suis* serotype 2 (SS2) by TLR2 and/or TLR4 (1). Similarly, CD11b/CD18, CD14, dectin-1, and TLRs recognize fungal mannans, leading to PKC activation and, ultimately, NOX-dependent NETosis (1, 9). NOX activation is achieved through PKC-mediated phosphorylation of its key subunits, such as p47^phox^ (10–12); indeed, p47^phox^ deficient B lymphoblasts fail to form an active NOX complex and consequently lack a reactive oxygen species (ROS) burst (13). This ROS burst is critical not only for bacterial killing by PMNs but also for initiating NETosis (11). ROS permeabilizes cellular membranes, including those of azurophilic granules that hold myeloperoxidase (MPO) and neutrophil elastase (NE), amongst other serine proteases (1, 4), which degrade cytoskeletal elements and thereby promote NETosis (3). These proteases also migrate to the nucleus, where they degrade lamin and histones, leading to chromatin decondensation and breakdown of the nuclear envelope (3, 14, 15).

Despite their crucial defensive role, excessive NET formation contributes to a range of pathologies. NET-associated molecules can act as autoantigens, triggering autoimmune responses. For example, citrullinated histones and vimentin serve as neoepitopes for anti-citrullinated protein antibodies in rheumatoid arthritis, NET-derived extracellular nucleic acids and dsDNA are targeted by autoantibodies in systemic lupus erythematosus, and NET-associated MPO and proteinase 3 are major autoantigenic targets in antineutrophil cytoplasmic antibody (ANCA)-associated vasculitis (1, 16). When these molecules escape from NETs, they can drive uncontrolled inflammation and tissue damage (1, 4, 17). In some SARS-CoV-2 infections, excessive NETosis is linked to acute lung injury (ALI) and acute respiratory distress syndrome (ARDS) through formation of a NET–IL-1β feedback loop, and in severe COVID-19, aberrant NET formation may also amplify cytokine storm and macrophage activation syndrome (18). SARS-CoV-2-mediated downregulation of angiotensin-converting enzyme 2 activates the High-Mobility Group Protein B1 (HMGB1) pathway, contributing to cytokine-storm-associated ALI/ARDS (18), and platelet-derived HMGB1, released during neutrophil–platelet interactions, can further induce inflammation and overstimulate NET formation. NETs themselves can also form a physical barrier that causes thrombosis and vascular obstruction, leading to organ damage such as brain injury in ischemic stroke, particularly when produced in excess (4, 18–20). Consequently, developing therapeutics that regulate or reduce excessive NETosis is critical for mitigating these complications.

HMGB1 is a versatile immune effector protein that is ubiquitously expressed among eukaryotes and performs diverse intracellular and extracellular functions. Intracellularly, HMGB1 functions as a DNA architectural element that facilitates DNA replication, transcription, repair, and chromatin organization (21, 22). HMGB1 binds and bends DNA, with high affinity for bent DNA structures such as Holliday junctions (HJ). Extracellularly, HMGB1 can act as a signal molecule where it can be internalized by endothelial cells through dynamin- and RAGE (receptor for advanced glycation end products)-dependent signaling (23). HMGB1 also acts as a proinflammatory innate immune effector that promotes PMN migration, induces NETosis, and exhibits anti-biofilm activity (24–26).

Biofilms are three-dimensional microbial communities that are either aggregated in suspension or adhered to the surface. Bacteria within biofilms produce a self-generated matrix that protects resident bacteria from environmental hazards, including host defenses and antimicrobials, while permitting free interaction among community members. Biofilm-resident bacteria are typically at least 1000-fold more tolerant of antibiotics than their isogenic planktonic counterparts (27), and biofilms are also resistant to PMN phagocytosis and NET-mediated clearance, making biofilm-associated infections extremely difficult to treat and resolve (28, 29). Although biofilm matrices contain species-specific structural components, extracellular DNA (eDNA) serves as a common structural element across biofilms (30, 31). We previously showed that recombinant HMGB1 (rHMGB1), through its DNA-binding activity, disrupts the eDNA-dependent protective matrix of biofilms, and thereby exhibits anti-biofilm activity (26). More recently, we showed that this anti-biofilm activity is localized to the B Box domain of HMGB1, which coincides with its proinflammatory activity (32). Notably, a synthetic variant of this domain, mB Box-97_syn_, engineered with a point mutation (C106S) to reduce its proinflammatory properties, retains DNA-binding and anti-biofilm activity (32). In a parallel study, we showed that this reduced proinflammatory activity was due in part to the ability of mB Box-97 to inhibit NET formation, through suppression of PKC activity and consequent reduction in NADPH oxidase (NOX2) activation (33).

Here, we constructed a series of short peptides derived from the Helix 1 region of mB Box-97, in the presence or absence of the adjoining linker region that connects the A Box and B Box domains of HMGB1 and introduced point mutations at key DNA-binding residues to further dissect peptide function. A 42-amino-acid peptide comprising the linker together with Helix 1, and bearing the C106S substitution, retained both the anti-NETotic and anti-biofilm activities of the parent peptide mB Box-97. As with mB Box-97, this linker–Helix 1 peptide suppressed PKC activity, thereby limiting NADPH oxidase activation and NETosis, while its intact DNA-binding capacity preserved anti-biofilm activity. Because many disorders characterized by excessive NETosis do not involve a biofilm component, we reasoned that unintended disruption of endogenous biofilms during NET-directed therapy could compromise host defenses. We therefore pursued two complementary strategies to uncouple these activities. First, truncating the linker region yielded a minimal 29-amino-acid Helix 1 peptide that retained anti-biofilm activity but lost the ability to inhibit NETosis, which indicated that the linker was dispensable for anti-biofilm function but was required, together with Helix 1, for anti-NETotic activity. Second, introducing DNA-binding mutations into the linker–Helix 1 peptide abolished its anti-biofilm activity while preserving its anti-NETotic activity, which confirmed that DNA binding underlies anti-biofilm function but was dispensable for PKC-dependent suppression of NETosis. Together, these complementary approaches allowed us to separate the anti-biofilm and anti-NETotic activities of mB Box-97 into distinct, minimal peptides, offering promising, readily synthesizable candidates for the selective control of biofilm- and NET-associated pathologies.

## 2 Methodology

### 2.1 Isolation of neutrophils from normal human blood

Blood was collected (20 mL per subject) in heparinized tubes (BD, Franklin Lakes NJ). Human neutrophils were isolated using the EasySepTM Human neutrophil isolation kit (StemCell Technologies, Inc., 17957) per manufacturer’s instructions. Isolated neutrophils were counted manually by hemocytometer.

### 2.2 Recombinant and synthetic proteins and peptides

Recombinant proteins without tags (A Box, B Box-97) were generated using the IMPACT kit (New England Biolabs). All constructs were confirmed by DNA sequencing. Proteins were purified to >95% purity using DEAE HiTrap and Heparin Sepharose columns and quantified using the Pierce BCA Protein Assay kit and SDS-PAGE with silver stain (ThermoFisher, 24612) with known protein standards. All recombinant proteins were tested for the presence of endotoxin using Pierce™ Chromogenic Endotoxin Quant Kit (Thermo Scientific™, A39552S) and endotoxin-free preparations were used for further studies. Proteins were also confirmed by Western blot analysis. 97 amino acids peptide mB Box-97_syn_, Helix1-C106 (H1C), Helix1-C106S (H1CS), Linker Helix1-C106 (LH1C), Linker Helix1-C106S (LH1CS), Helix1,2-C106 (H1,2C), Helix1,2-C106S (H1,2CS), Linker Helix1,2-C106S (LH1,2CS), Linker Helix1-C106S carrying an R97A mutation (LH1CS-RA), and Linker Helix1-C106S carrying an F103Y mutation (LH1CS-FY), were synthesized by LifeTein, LLC, 601 US Rt.206, Suite 26–463 Hillsborough, NJ 0884 (LifeTein^®^, LLC; for ease of large-scale production and > to 95% homogeneity). Sequences of all peptides are provided in **Supplementary Data Sheet 1**. R97 and F103 were selected for mutagenesis because they are established DNA-contact residues within the HMGB1 B Box that mediate DNA binding and intercalation, respectively (34, 35); substituting these residues (R97A, F103Y) was therefore expected to selectively disrupt DNA-binding activity while leaving the peptide backbone otherwise intact.

### 2.3 Quantitation of NETs

10^5^ PMNs per well in Hanks’ Balanced Salt Solution (HBSS) supplemented with calcium and magnesium (Fisher, 14025092) in an 8-well glass bottom chambered slide were allowed to attach for 30 minutes at 37 °C. Cells were then stimulated with 100 nM Protein Kinase C (PKC) activator phorbol 12-myristate 13-acetate (PMA) (Sigma Aldrich, P1585) with or without peptides B Box-97/mB Box-97_syn_/LH1C/LH1CS/LH1CS-FY or LH1CS-RA (200 nM) and incubated for 3–4 hours at 37 °C. DNA was stained with 5×10^−6^ M SYTOX™ Green (Invitrogen, S7020) for 15 min. NETs were imaged with a Zeiss LSM 800 confocal microscope (Carl Zeiss Inc.). Cells were counted manually and % NET forming cells of the total number of cells present per image were plotted using GraphPad Prism.

10^5^ PMNs were treated with either media alone or 100 nM PMA in the presence or absence of the above peptides or peptides alone for 3 hours. Extracellular DNA was stained with 5×10^−6^ M of SYTOX™ green (Invitrogen, S7020) for 15 minutes, and sample fluorescence was measured at 504/523 nm with a Synergy H1 multi-mode plate reader.

### 2.4 Assessment of biofilm disruption

Non-typeable *Haemophilus influenzae* (NTHI) and Methicillin-Resistant Staphylococcus aureus (MRSA) were cultured on chocolate agar for 18-24h at 37 °C in a humidified atmosphere that contained 5% CO_2._ NTHI was then resuspended in brain heart infusion broth (BHI) supplemented with heme (2 μg/mL) and β-NAD (2 μg/mL) (sBHI) broth to an OD_490_ of 0.1. MRSA was resuspended in BHI to an OD_490_ of 0.1. *P. aeruginosa* was cultured on tryptic soy agar (TSA), then suspended in tryptic soy broth (TSB) to an OD_490_ of 0.1. *K. pneumoniae* was cultured on LB agar for 18–24 h at 37 °C in a humidified atmosphere that contained 5% CO_2_; then suspended in Todd Hewitt broth (THB) to an OD_490_ of 0.1. *S. pneumoniae* was cultured on blood agar (TSA with sheep blood), then suspended in Todd Hewitt broth (THB) to an OD_490_ of 0.1. Cultures were then diluted in their respective medium to contain approximately 2 × 10^5^ CFU/mL, and 200 μL of this suspension was inoculated into each well of an 8-well chambered cover-glass slide (Thermo Fisher Scientific, Waltham, MA). After 16h of incubation of each bacterial species at 37 °C 5% CO_2_, medium was replaced with the respective fresh medium and incubated for an additional 8h. After a total incubation period of 24h, medium was replaced with the respective fresh medium (control) or fresh medium that contained, A Box or mB Box-97_syn_/H1C/H1CS/LH1CS/LH1C/LH1CS-RA or LH1CS-FY (200 nM) and incubated at 37 °C 5% CO_2_ for 2 hours. All biofilms were then washed twice with 1× Dulbecco’s phosphate-buffered saline without calcium or magnesium (DPBS) (Corning, Corning, NY) and stained with LIVE/DEAD^®^ stain (Thermo Fisher Scientific, Waltham, MA) per manufacturer’s instructions. Biofilms were again washed then fixed with 1.6% paraformaldehyde, 0.025% glutaraldehyde, and 4% acetic acid in 0.1 M phosphate buffer at pH 7.4. Biofilms were imaged with a 63× objective on a Zeiss 800 confocal laser scanning microscope (CLSM; Zeiss) wherein image selection was determined through analysis of the distribution of biofilm growth within chamber slide wells that best represented the entirety of each well, with areas in the proximity of where we introduced pipet tips and/or sides of chamber slide walls avoided. Images were analyzed with COMSTAT2. Biomass values (μm^3^/μm^2^) were calculated by COMSTAT2 and represent the mean of 3 biological replicates that were repeated a minimum of three times on separate days (n≥3).

### 2.5 Visualization of NET structures

1 x 10^5^ PMNs in HBSS (Fisher, 14025092) per well in 8-well glass bottom chambered slide were allowed to attach for 30 minutes at 37 °C. Cells were then stimulated with 100 nM PMA, heat-inactivated NTHI (MOI = 10), or 1 µM Ca2+ ionophore A23187 (Millipore Sigma, C7522) with or without peptides B Box-97/mB Box-97_syn_/LH1C/LH1CS/LH1CS-FY or LH1CS-RA (200 nM) and incubated for 3–4 hours at 37 °C. To visualize NETs, we used a method previously described (26). Briefly, NETs were fixed in formalin, blocked with 10% normal goat serum (Fisher, 50197Z), and labeled with α-dsDNA mouse monoclonal antibody (1 μg; Abcam, ab27156), and α-neutrophil elastase rabbit monoclonal antibody (1 μg; Abcam ab131260) or anti-myeloperoxidase recombinant rabbit monoclonal antibody (1 μg; Abcam, ab5103) or naive rabbit and mouse isotype controls (1 μg; Abcam, ab37415 and ab18415) in 200 μl PBS for 16 hours at 4°C. NETs were washed with PBS and incubated for 1 hour with 1:200 dilution goat α-rabbit IgG conjugated to AlexaFluor^®^ 405 (Invitrogen, A11032), goat α-mouse IgG conjugated to AlexaFluor^®^ 594 (Invitrogen, A11001), and 1:500 dilution wheat germ agglutinin conjugated to AlexaFluor^®^ 488 (PMN membrane stain, Fisher scientific, W6748). NETs were imaged with a Zeiss LSM 800 confocal microscope (Carl Zeiss Inc.) and images rendered with Zeiss Zen software. Using ImageJ, Z-stack projection of the images was generated by summing all the stacks, followed by brightness and contrast adjustments using the auto setting for all fluorophores to improve the image quality of the representative images presented in this manuscript. Mean Fluorescence Intensity (MFI) values for NE/MPO, dsDNA, and Plasma Membrane (PM) were quantified using ImageJ.

### 2.6 Quantification of released and DNA-bound NE

A total of 5 x 10^6^ neutrophils in HBSS (Fisher, 14025092) per well were seeded into 6-well plates. Cells were treated with media alone or 200 nM PMA with or without peptides B Box-97/mB Box-97_syn_/LH1C/LH1CS/LH1CS-FY/LH1CS-RA (1 μM) for 3–4 hours. Media from each well was collected and centrifuged at 10,000 g for 10 min to remove the cells or cell debris. This fraction was labeled as secreted NE. The NETs at the bottom of each well were incubated with DNase I for 30 min, cells, and cell debris were removed by centrifugation as mentioned above and the supernatant collected was labeled as DNA-bound. Both fractions were subjected to the quantification of NE using a Human Neutrophil Elastase/ELA2 DuoSet ELISA kit (R&D systems, DY9167-05) per manufacturer’s instructions. Briefly, the capture antibody was coated to the 96 well plates overnight. Samples and standards were incubated for 2 hours at RT and washed thrice with wash buffer post incubation. Each well was incubated with detection antibody for 2 hours at RT and washed twice post incubation with wash buffer. The detection antibody was probed with streptavidin-HRP by incubating at RT for 20 min. Post incubation each well was washed twice with wash buffer and incubated for 20 min (or till the color develops) at RT, the reaction was stopped with 2 N H_2_SO_4_ (stop solution) and plates were read using a plate reader.

### 2.7 Measurement of reactive oxygen species

PMNs were stained with highly reactive oxygen species (hROS) detection reagent Luminol (36) as described (37). Briefly, 10^5^ PMNs were seeded into the wells of 96 well black plates and incubated for 10 min at 37 °C. After the addition of 100 µM luminol (Sigma Aldrich) cells were incubated for 5 min at RT, and activated with 100 nM of PMA alone, PMA with peptides B Box-97/mB Box-97_syn_/LH1C/LH1CS/LH1CS-FY/LH1CS-RA (200 nM) or media alone. Luminescence as a measure of generation of ROS was measured for 120 min at 5 min intervals using Synergy H1 multi-mode plate reader (Biotek, Winooski VT).

### 2.8 Western blot analysis

5 x 10^6^ isolated human neutrophils were treated with media alone (HBSS, Thermo Scientific, 14025092) or PMA (200 nM) with or without peptides B Box-97/mB Box-97_syn_/LH1C/LH1CS/LH1CS-FY or LH1CS-RA (1 µM) for 30min. Cells were homogenized in RIPA Lysis and Extraction Buffer (Thermo Scientific, 89901) supplemented with Halt™ Protease and Phosphatase Inhibitor Cocktail (Thermo Scientific, 78442). The concentration of total proteins was quantified using the BCA (Thermo Scientific, 23227) and, 20 µg total proteins were separated on 12% SDS-PAGE gels and electro-transferred to nitrocellulose membranes, blocked for 30 min with 5% fat free dry milk in PBST (PBS with 0.1% Tween 20) before the primary antibodies were applied. Antibodies used in Western blot were anti phospho-p47^phox^ (Ser370) rabbit polyclonal antibody (1:500, Invitrogen, PA5-36863), rabbit monoclonal anti p47^phox^ (Abcam, EPR27205-231) Mouse anti-GAPDH (1:6000, Proteintech, 60004-1-Ig), HRP-conjugated anti-rabbit (1:10,000, Jackson, 111-035-003), HRP-conjugated anti-Mouse (1:10,000, Jackson, 115-035-003). After overnight incubation at 4°C with primary antibodies and washing, corresponding HRP-conjugated secondary antibodies were incubated for 2 h at RT, followed by washing. HRP signals were detected by an enhanced chemiluminescence reagent (Thermo Scientific, PI80196) and imaged by a super-sensitive multifunctional imaging instrument (Amersham Imager 680UV, GE). Density of each protein band was analyzed using ImageJ and the density of phospho p47^phox^ was plotted post normalization with total p47^phox^ and GAPDH as Arbitrary Units (AU) using GraphPad Prism.

### 2.9 Inactivation of NET mediated bacterial killing

Inactivation of NETs-mediated bacterial killing was measured as described previously (31). Briefly, NTHI strain 86-028NP, isolated from the nasopharynx of a child with chronic otitis media at Nationwide Children’s Hospital (38) was cultured on chocolate agar for 18-24h at 37 °C in a humidified atmosphere that contained 5% CO_2_. NTHI was then resuspended in brain heart infusion broth supplemented with heme (2 μg/mL) and β-NAD (2 μg/mL) (sBHI) broth to an OD_490_ of 0·1. The culture was then diluted to approximately 2 × 10^5^ CFU/mL, and 200 μL of this suspension was inoculated into each well of an 8-well chambered cover-glass slide (Thermo Fisher Scientific, Waltham, MA) for 16 hours. Biofilms were washed carefully with PBS to remove non-adherent bacteria, followed by the addition of 300 ml of RPMI supplemented with 10% FBS. 1x10^6^ neutrophils were then added to the wells and treated with 1 µM HU_NTHI_ (positive control), 1 µM B Box-97/mB Box-97_syn_/LH1C/LH1CS/LH1CS-FY or LH1CS-RA for 3–4 hours. After incubation, during which time biofilm remodeling occurs, 0.1% Triton X-100 was added to the cultures to release any intracellular bacteria from the PMNs after which all bacteria recovered by homogenization via pipetting, followed by serial dilutions and plating (37). The percentage of reduction of the number of NTHI (total CFU) and % relative killing was calculated as indicated in the following formulas:

(CFU of each sample) ÷ (average CFU of NTHI only controls) *100 = % viable

% Killing = 100% - (% viable)

For each biological replicate, % killing was calculated relative to that replicate’s own paired NTHI-only (no-neutrophil) control, which was thereby defined as 0% killing; conditions with fewer viable bacteria than this control therefore yield correspondingly higher % killing values, as follows:

relative % killing = 100 × [1 − (CFU of sample ÷ mean CFU of the paired NTHI-only control)]

The graph plots the data of the PMNs derived from 6 healthy donors ± SEM. The statistical analysis was performed with One-way ANOVA and Dunnett multiple comparison test. (** = P < 0.01).

### 2.10 Inhibition of PKC activity

The effect of B Box-97/mB Box-97_syn_/LH1C/LH1CS/LH1CS-FY or LH1CS-RA on the activity of PKC was assessed using a PKC kinase activity kit (Enzo Life Sciences, ADI-EKS-420A) as per manufacturer instructions. Briefly, 30 µL of 2 ng PKC with or without proteins (histone H1 (positive control), B Box-97(negative control) or mB Box-97_syn_/LH1C/LH1CS/LH1CS-FY or LH1CS-RA) at 1µM final concentration in the activity assay buffer were added to the precoated wells with PKC substrate. The reaction was initiated using 10 µL ATP and incubated at 30°C for 90 min. After the removal of reaction reagents, each well was incubated with 40 µL of the phospho-specific antibody for 60 min at RT. Each well was washed 4 times with wash buffer and 40 µL of the Anti-Rabbit IgG: HRP conjugate was added to each well, except the blank. After incubation for 30 min at RT, each well was washed with wash buffer 4 times. 60 µL TMB substrate was added to each well and incubated at RT till the development of the color (30–60 min). The reaction was stopped by adding 20 µL of the stop solution and read at 450 nM using a plate reader. Data was normalized and plotted using GraphPad Prism.

### 2.11 Glutaraldehyde crosslinking assay

Crosslinking was performed as previously described with slight modifications (39). Briefly, PKC alone or PKC and mB Box-97_syn_/LH1C/LH1CS/LH1CS-FY or LH1CS-RA at 1:2 molar ratio was incubated with 0.01% glutaraldehyde in HBSS (Thermo Scientific, 14025092) and 5 µL 1 mg/mL ATP for 30 minutes at room temperature in a total reaction volume of 15 µL. Equal volumes of 2× Laemmli sample buffer were added, and the samples were separated using SDS-PAGE 12% gel. Gels were either stained with silver stain (ThermoFisher, 24612) or transferred onto nitrocellulose paper to investigate the presence of PKC using anti-PKC alpha rabbit antibody (Cell Signaling, 2056S) which was then detected using anti HRP-conjugated anti-rabbit (1:10,000, Jackson, 111-035-003). HRP signals were detected by an enhanced chemiluminescence reagent (Thermo Scientific, PI80196) and imaged by a super-sensitive multifunctional imaging instrument (Amersham Imager 680UV, GE).

### 2.12 Stability assessment of peptides

1µg mB Box-97_syn_ was incubated with human serum or PBS for either 0 or 24 hours. To test the stability of the peptide in neutrophil conditioned media, a total of 5 x 10^6^ neutrophils in HBSS (Fisher, 14025092) per well were seeded into 6-well plates. Cells were treated with 200 nM PMA for 4 hours. Media was collected and centrifuged at 10,000 RPM for 10 min to remove the cells or cell debris. 1µg mB Box-97_syn_/LH1C/LH1CS/LH1CS-FY/LH1CS-RA was incubated with the conditioned media from above preparation or HBSS alone for 4 hours. Post incubations the peptides were separated by 16.5% Tris-Tricine gel (Bio-Rad, 4563066) and stained using silver stain (Thermo Scientific, 24612).

### 2.13 Electrophoretic mobility shift assay

HJ DNA (J12) was generated as described previously (40, 41) with oligos 1 (5’-GACGCTGCCGAATTCTGGCTTGCTAGGACATCTTTGCCCACGTTGACCC – 3’, 2 (5’ - 6-carboxyfluorescein (FAM) – TGGGTCAACGTGGGCAAAGATGTCCTAGCAATGTAATCGTCTATGACGTT – 3’), 3 (5’ –CAACGTCATAGACGATTACATTGCTAGGACATGCTGTCTAGAGACTATCGA – 3’), 4 (5’- ATCGATAGTCTCTAGACAGCATGTCCTAGCAAGCCAGAATTCGGCAGCGT – 3’), and 5 (5’ – AACGTCATAGACGATTACATTGCTAGGACATCTTTGCCCACGTTGACCCA – 3’). Equimolar concentrations of the respective oligos 1, 3, 4, and 5 (50 μM) and oligo 2 (25 μM) were mixed in a reaction that contained 10 mM Tris-HCl, pH 7.8, 100 mM NaCl and heated to 95 °C for 10 minutes, 65 °C for 10 minutes, 37 °C for 10 minutes, and 20 °C for 10 minutes. The HJ, Y, and duplex DNA were then resolved by 6% non-denaturing polyacrylamide gel electrophoresis in 0.5X TBE running buffer at 200 Volts for 2.5 hours and were purified from the gel by crush and soak method. RuvA (5 and 10 μM) was incubated with 7.5 nM HJ-DNA in EMSA reaction buffer (50 mM Tris pH 7.8, 50 mM KCl, 100 μg/ml BSA, 200 μM EDTA, 1 mM DTT) for 30 minutes at 20 °C. Binding of mB Box-97_syn_ and Helix-1 peptides (LH1CS, LH1C, LH1CS-RA, LH1CS-FY) (100 and 200 nM) were incubated with 20 nM HJ DNA, duplex in EMSA reaction buffer for 30 minutes at 20 °C. The reaction mixtures were then separated by 6% non-denaturing polyacrylamide gel electrophoresis in 0.5X TBE running buffer at 200 Volts for 2.5 hours. The resolved DNA was imaged with a FluorChem M FM0470 (Azure Biosystems). The assay was repeated three times on separate days.

### 2.14 Peptide treatment with DTT and NTHI disruption and NETosis assay.

LH1C (1μg/200nM) peptides were treated with 1mM DTT for 5/10/15 and 30 minutes before separating on 16.5% Tris-Tricine gel (Bio-Rad, 4563066) LH1CS was used as a negative control and treated with DTT at 1mM for 30 minutes. LH1C/LH1CS (200nM) were treated with 1mM DTT for 30 minutes and then used for disruption of 24 hour old NTHI biofilms, DTT alone was used as a control. Both these peptides were used to assess their effect on PMA induced NETosis in presence or absence of 1mM DTT using confocal assay and plate-based assay using SYTOX™ green (Invitrogen, S7020) as mentioned above.

### 2.15 Statistics

Graphical results were analyzed, and statistical tests were performed with GraphPad Prism 10. Data from two groups were analyzed by unpaired t-tests, whereas data from multiple groups were analyzed by one-way/two-way analysis of variance (ANOVA). Data are presented as mean ± SEM and significance is shown as *P< 0·05, **P < 0·01, ***P < 0·001, and ****P <0·0001. All the statistical details are provided in the respective Figure legends of each Figure which include the statistical tests used, the exact value of n, and what n represents. Each biological replicate had 3 technical replicates.

## 3 Results

### 3.1 Helix based peptides derived from mB Box-97_syn_ inhibit NETosis by human neutrophils

In a previous study (33), we described the anti-NETotic activity of a synthetic mutant peptide, mB Box-97_syn_ which is based on B Box domain of HMGB1, (NCBI-BAA09924.1) except for a single amino acid change where a Cysteine is mutated to Serine at the 106 amino acid position in HMGB1. This C106S mutation enabled mB Box-97_syn_ to inhibit the PKC activity and subsequently suppress NET formation in isolated human neutrophils *in vitro*. Because the full-length 97-amino-acid peptide was difficult to synthesize at scale, we designed smaller peptide fragments to identify the location of the anti-NETotic activity. mB Box-97_syn_ contains a positively charged linker region (13 amino acids) connecting the A Box and B Box and three alpha helices (**Figure 1A**). Since the anti-NETotic activity is attributed to the C106S mutation, we generated peptides that contained either helix 1 alone or helices 1 and 2, each with or without the linker sequence that connected the A Box and B Box domains of HMGB1. These constructs were named Helix1-C106 (H1C), Helix1-C106S (H1CS), Linker Helix1-C106 (LH1C), Linker Helix1-C106S (LH1CS), Helix1,2-C106 (H1,2C), Helix1,2-C106S (H1,2CS), Linker Helix1,2-C106S (LH1,2CS). Sequences for all constructs are provided in **Supplementary Data Sheet 1**.

**Figure 1:**
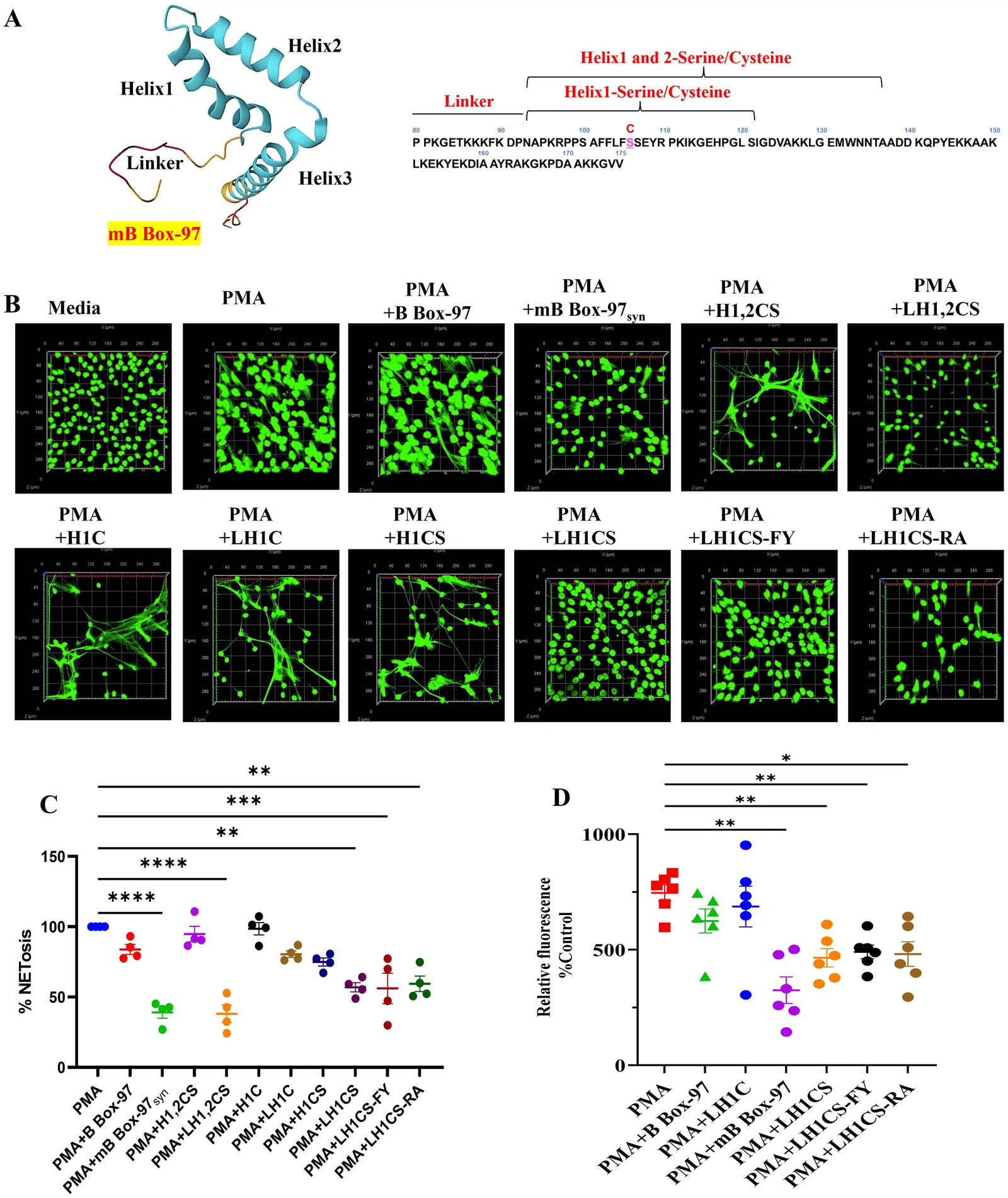
Helix 1 based peptides derived from mB Box-97_syn_ inhibited PMA induced NETosis. **(A)** 3D structure of mB Box-97_syn_ predicted using ESMFold2 online algorithm (https://biohub.ai/tools/fold) (42) depicting 3 alpha helices and linker region. Corresponding amino acid sequence of mB Box-97_syn_ is shown with Linker region, Helix 1 and Helix1 and 2 are shown. Specific Cysteines/C amino acid position is shown in red and its mutation to Serine/S is shown in pink at position 106. **(B)** NETs were induced using PMA in the presence or absence of respective peptides (200nM) and stained to detect DNA using SYTOX Green (green). NETs were visualized using CSLM at 20x magnification, with representative images provided for each treatment condition out of 4 independent biological replicates (n). **(C)** Formed NETs were manually quantified using ImageJ, and the percentage of NET formation was calculated relative to total PMNs in each image area. Mean values of n = 4 biological replicates ± Standard error of the mean (SEM) is shown. **(D)** Fluorescence of DNA released from the PMNs induced with PMA with or without the addition of peptides (200nM) was plotted as a percentage relative fluorescence compared to the untreated control group. Mean values of n = 6 biological replicates ± SEM are shown. P values (ns = P > 0.05 (not shown), *P < 0.05, **P < 0.01, ***P < 0.0005, ****P < 0.0001) are calculated using a Welch and Brown-Forsythe ANOVA followed by Dunnett’s T-test.

Human neutrophils (PMNs) were incubated with each peptide, fixed, permeabilized, and stained for DNA using SYTOX Green. Cells were imaged by CSLM, and NET-positive cells were quantified. Only constructs that contained both the linker sequence and the C106S mutation significantly inhibited PMA-induced NETosis (**Figures 1B, C**). Although Linker-Helix1,2-C106S (LH1,2CS) also significantly inhibited NETosis, we prioritized the smaller Helix-1-only constructs (LH1CS) for all subsequent experiments because they retained comparable anti-NETotic activity while being simpler and more cost-effective to synthesize at the scale required for further mutagenesis and downstream functional assays. We also confirmed this inhibitory effect by a plate-based assay, in which extracellular DNA released during NETosis was stained with SYTOX Green and quantified by fluorescence relative to media-only control (**Figure 1D**).

To confirm that the peptides remained intact throughout the experiments, we assessed their stability under two conditions: conditioned media from PMA-stimulated neutrophils undergoing NETosis for 6 hours, and 10% human serum in PBS. Peptides were incubated for 4 hours (the longest incubation time with neutrophils in our assays) with neutrophil conditioned media and 24 hours with 10% human serum, then analyzed by SDS-PAGE to detect degradation. After 4 hours in neutrophil-conditioned media, peptides showed minimal degradation (<8-15%) while there was no detectable (< 6-9%) degradation observed upon incubation in 10% human serum (**Supplementary Figure S1**). Together, these results show that the helix-based peptides carrying the C106S mutation and linker sequence inhibited PMA-induced NETosis in human neutrophils as effectively as the full-length mB Box-97_syn_ peptide, thereby confirming our hypothesis that the cysteine-to-serine substitution is essential for anti-NETotic activity of these peptides. These observations also show that a minimal peptide fragment is sufficient for anti-NETotic activity.

### 3.2 DNA binding mutants significantly reduced the biofilm inhibition by LH1CS peptide

mB Box-97 also exhibits anti-biofilm activity attributable to its DNA-binding capacity (32), therefore we tested all the peptide constructs for their ability to disrupt biofilms. Towards that goal peptides H1C; H1CS; H1,2C; H1,2CS; LH1CS; or LH1,2CS were incubated with 24 hr NTHI biofilms, stained with LIVE/DEAD^®^, imaged by CLSM then analyzed by COMSTAT2 to calculate biofilm biomass. All peptides were able to disrupt NTHI biofilms (**Supplementary Figure S2**).

Many excess NETosis related ailments lack a biofilm component. Therefore, we determined if we could create a peptide derivative that lacked antibiofilm activity but retained anti-NETotic function. To address this issue, we generated two additional peptides on the LH1CS backbone, LH1CS-FY with F103Y mutation and LH1CS-RA with R97A mutation of the known DNA-binding residues within the helix 1 region of HMGB-1 B Box (34, 35) (**Supplementary Data Sheet 1**). To validate that these two constructs had lower DNA binding activity, we performed an electromobility shift assay (EMSA) using a HJ DNA substrate that mimics the crossed strands of the eDNA lattice of bacterial biofilms (43) which is also a natural substrate of HMGB1 (26). LH1CS peptide showed comparable HJ DNA binding to mB Box-97_syn_ (**Figure 2A**). In contrast, LH1C; LH1CS-FY and LH1CS-RA lacked HJ DNA binding (**Figure 2A**). Introduction of these mutations failed to affect the anti-NETotic activity (**Figures 1B–D**), nor did they affect the stability of these peptides (**Supplementary Figure S1**). The lack of DNA binding of LH1C peptide can be attributed to the intermolecular disulfide bond formation between two LH1C monomers (**Supplementary Figure S3A**). The LH1C dimer could be returned to the monomeric state using DTT (1mM) for more than 15min (**Supplementary Figure S3A**) and that monomeric form was able to bind to HJ DNA (**Supplementary Figure S3B**). Moreover, the reduced monomer of the LH1C peptide was able to disrupt NTHI biofilms (**Supplementary Figure S4**) with no measurable effect of DTT on NTHI biofilm or antibiofilm activity of LH1CS peptide (**Supplementary Figure S4**). When tested, DTT did not show any effect on the activity of LH1C or LH1CS peptide when tested against PMA induced NETosis (**Supplementary Figure S5A, B**).

**Figure 2:**
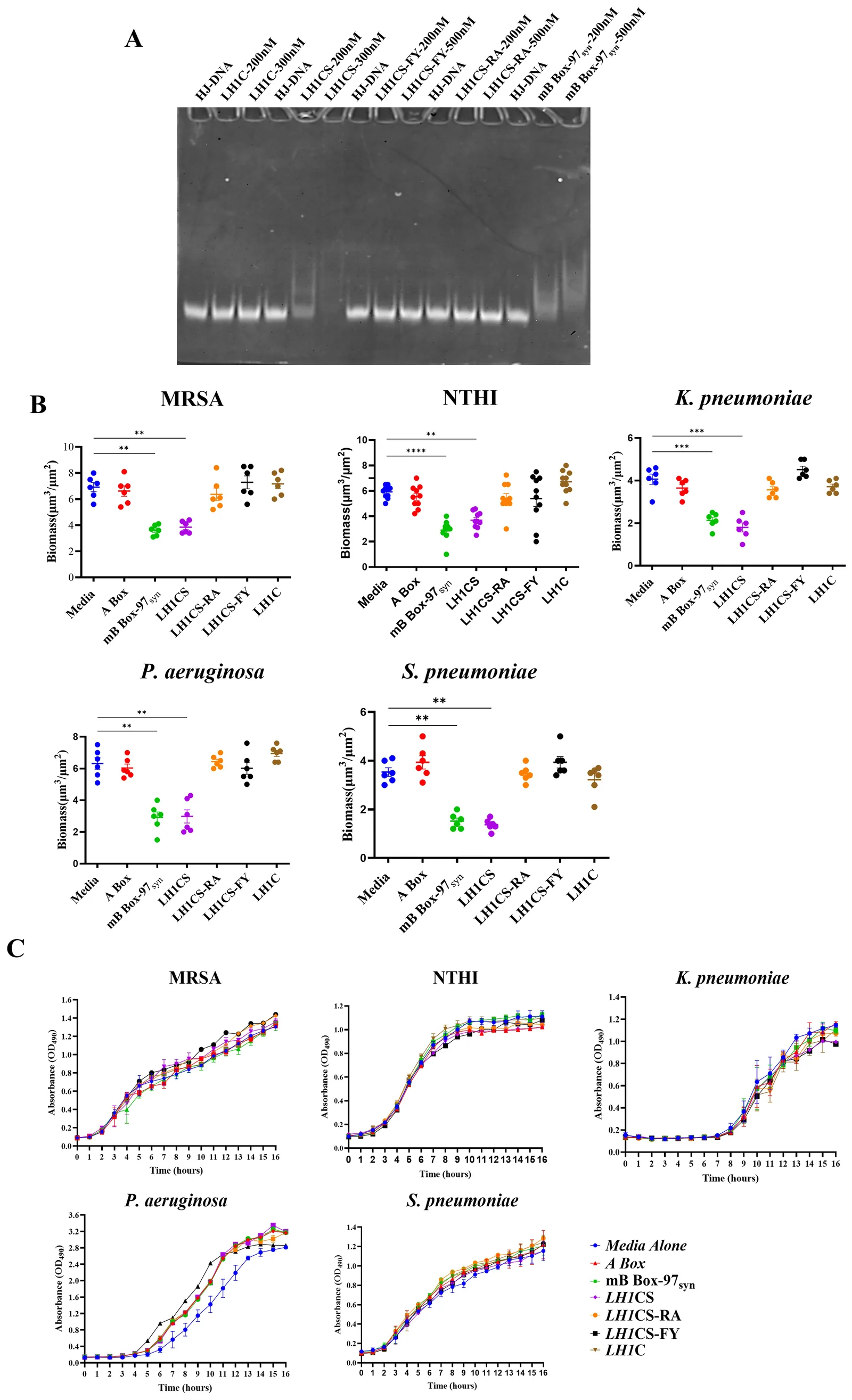
Introduction of DNA binding mutation inhibited the biofilm disruption ability of LH1CS peptide. **(A)** Electrophoretic Mobility Shift Assay (EMSA) of mB Box-97_syn_ and Helix-1 peptides. Increased concentrations of mB Box-97_syn_, LH1CS, LH1C, LH1CS-RA, or LH1CS-FY incubated with HJ DNA are shown. Complexed HJ DNA with Helix-1 peptides demonstrated a slower relative migration pattern to free HJ DNA. **(B)** Helix-1 peptide variants were assayed to evaluate anti-biofilm activity. The biomass content of pre-formed by NTHI, MRSA, *K. pneumoniae, P. aeruginosa*, and *S. pneumoniae*, *in vitro*, treated with Media alone or with A Box/mB Box-97_syn_/LH1CS/LH1C/LH1CS-RA or LH1CS-FY (200 nM) for 2h is shown. Data presented are mean ± SEM., N = 3. **(C)** Growth of above-mentioned pathogens in the presence or absence of peptides (200nM) was monitored for 16 h and plotted as a measure of OD at 490, N = 3. Statistical significance compared to media alone control was assessed with one-way ANOVA using nonparametric Kruskal–Wallis test (with an uncorrected Dunn’s test for multiple comparisons) (*P < 0.01, **P < 0.01, ***P < 0.001, and ****P < 0.0001). Due to the lack of DNA binding peptides LH1CS-RA and LH1CS-FY lost anti-biofilm activity.

In our previous publication, we showed that mB Box-97_syn_ binds to HJ DNA and thereby disrupts biofilms (32). Due to their lack of DNA binding, we hypothesized that both LH1CS-FY and LH1CS-RA would lose their anti-biofilm activity. To this end, we assayed for the ability of the two aforementioned peptides to disrupt biofilms formed by NTHI, MRSA, *P. aeruginosa, K. pneumoniae*, or *S. pneumoniae*. Negative controls included exposure to media alone, A Box (200 nM) (26, 32), or LH1C peptide (200 nM). LH1C, which retains the native (unmutated) cysteine at position 106, was included as a control specifically for the C106S substitution itself, distinct from LH1CS-RA and LH1CS-FY, which were purpose-built with targeted DNA-contact mutations to test the DNA-binding-dependent model of anti-biofilm activity; LH1C was therefore not designed, nor is it used here, as a DNA-binding-deficient comparator. Established biofilms (24 h) were treated with respective peptides for 2 h and assayed for relative disruption. Biofilms were stained with LIVE/DEAD^®^ stain, imaged by CLSM then analyzed by COMSTAT2. Neither exposure to negative control A Box, nor LH1CS-RA or LH1CS-FY disrupted established biofilms formed by any of the 5 tested pathogens (**Figure 2B**). Against all pathogens tested, mB Box-97_syn_ and peptide LH1CS disrupted preformed biofilms (**Figure 2B**). As LH1C lacked DNA binding, it also was unable to disrupt biofilms. Importantly, none of the peptides inhibited growth of any of these pathogens which indicated that the anti-biofilm effect was not due to direct bacteriostatic or bactericidal activities (**Figure 2C**). By introducing DNA binding mutations, we created two distinct peptides which lacked anti-biofilm activity thereby successfully isolating anti-NETotic activity in LH1CS-RA and LH1CS-FY peptides whereas the peptides that lacked the linker sequence (H1CS) possessed only anti-biofilm activity.

Henceforth, we will refer to mB Box-97 Helix-1 based peptides (LH1CS/LH1CS-FY/LH1CS-RA) collectively as Helix-1 peptides.

### 3.3 Helix-1 peptides inhibited release of NETs-associated proteins

Since Helix-1 peptides showed anti-NETotic activity morphologically, we investigated their impact on the release of NETs-associated proteins such as NE and MPO during peptide-mediated inhibition of NETosis. Isolated human PMNs were treated with PMA with or without B Box-97/mB Box-97_syn_/LH1C/LH1CS/LH1CS-FYor LH1CS-RA and stained with specific antibodies to identify DNA, the plasma membrane (PM), and NET-associated proteins (NE/MPO). NET formation was observed by comparing z-stack images captured by CSLM. Helix-1 peptides inhibited PMA-induced NETs formation to a degree comparable to mB Box-97_syn_ (**Figures 3A, B**), whereas B Box-97 or LH1C had no effect.

**Figure 3:**
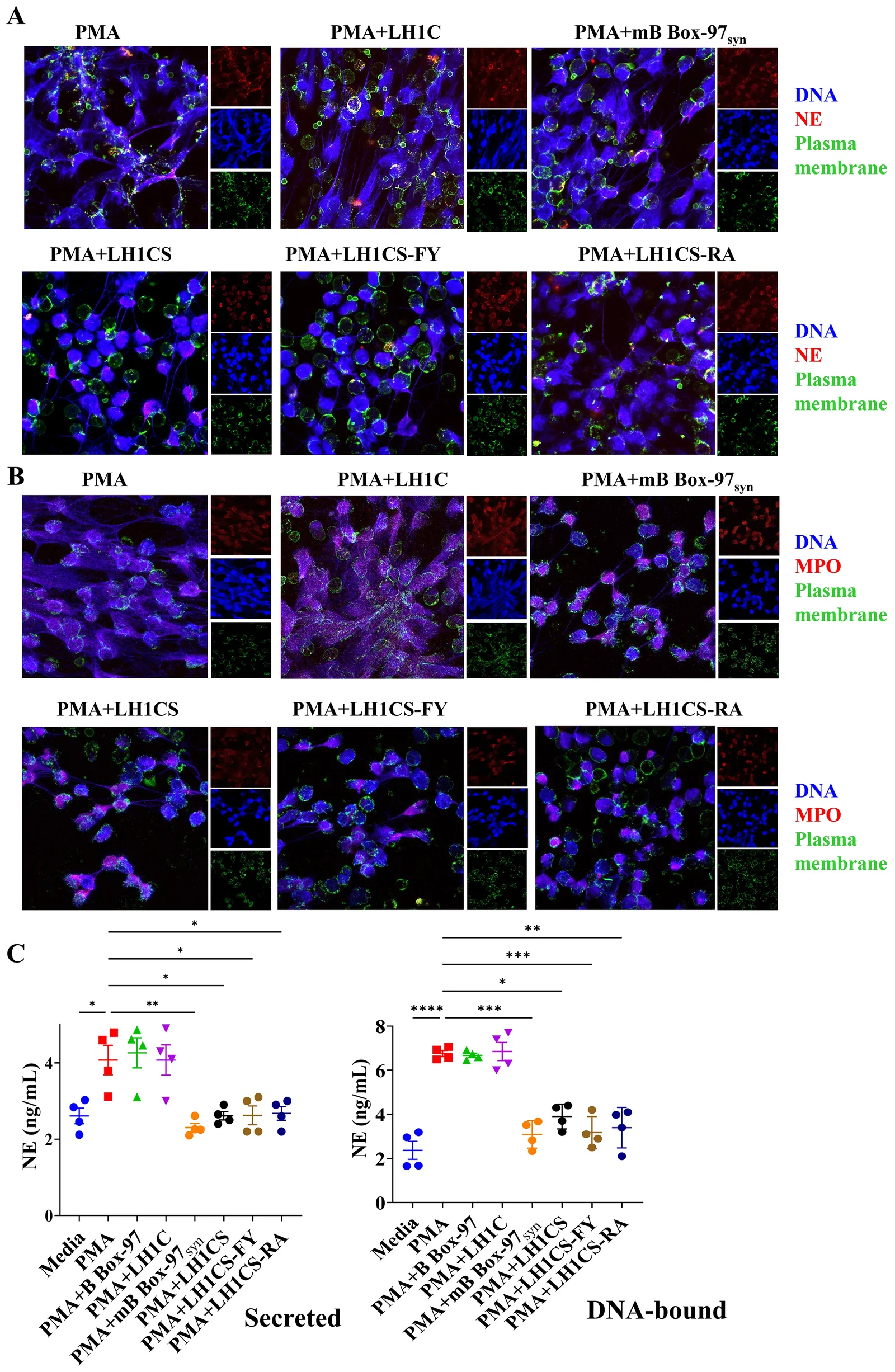
Helix-1 peptides inhibited PMA induced NETosis as well as the secretion of the NET-associated protein Neutrophil Elastase (NE). The induction of NETs by PMA was assessed in isolated human PMNs in the presence or absence of respective peptides (200nM). NETs were stained for NET-associated proteins, namely **(A)** Neutrophil Elastase (NE) and **(B)** Myeloperoxidase (MPO) shown in red; DNA shown in blue and plasma membrane shown in green. Representative CLSM images for each treatment condition are shown out of 3 independent experiments (n). **(C)** ELISA to detect secreted or DNA bound NE. PMNs were induced to form NETs with PMA alone or PMA +/- peptides (1µM) and NE released (secreted) into the media was gathered. NETs settled at the bottom of respective well were treated with DNase I to release DNA-bound NE (DNA-bound). The concentration of NE was quantified using ELISA and presented as mean ± SEM of n = 4 biological replicates. P values (ns = P > 0.05, *P < 0.05, **P < 0.005, ***P < 0.001, and ****P < 0.0001) using one way ANOVA followed by Tukey’s multiple comparison test.

We calculated the MFI for NE/MPO; DNA and PM. MFI values for NE/MPO were normalized to the MFI for DNA and PM. Addition of mB Box-97_syn_ and Helix-1 peptides reduced the release of NE and MPO, as indicated by these normalized MFI values (**Supplementary Figure S6**). We further examined NE release into the surrounding medium and NE bound to DNA following PMA-induced NETosis. Neutrophils were stimulated to form NETs, and DNA-bound and secreted NE were quantified using an NE-specific ELISA kit. Free (secreted) NE contributes to chronic inflammation, whereas DNA-bound NE within NETs degrades plasminogen, reduces plasmin formation, and decreases fibrinolysis (44, 45). Treatment with Helix-1 peptides significantly reduced both secreted NE and DNA-bound NE (**Figure 3C**).

### 3.4 Helix-1 peptides bind to PKC and inhibit PKC activity

In our previous publication, we showed that mB Box-97_syn_ binds to PKC. We also demonstrated that the introduced serine mutation generates a new PKC phosphorylation site (33) which results in the inhibition of PKC and downstream NETosis. Because Helix-1 peptides contain this same mutation and also showed anti-NETotic activity comparable to mB Box-97_syn_, we hypothesized that Helix-1 peptides share a similar mode of action with mB Box-97_syn_. Towards that goal, we used a PKC activity assay kit to assay whether Helix-1 peptides could inhibit PKC kinase activity. mB Box-97_syn_ and Histone H1, a known PKC substrate (46), were used as positive controls for PKC inhibition. Indeed, as shown in **Figures 4A** Helix-1 peptides inhibited PKC activity, whereas LH1C, which lacks the serine mutation, had no effect on PKC activity.

**Figure 4:**
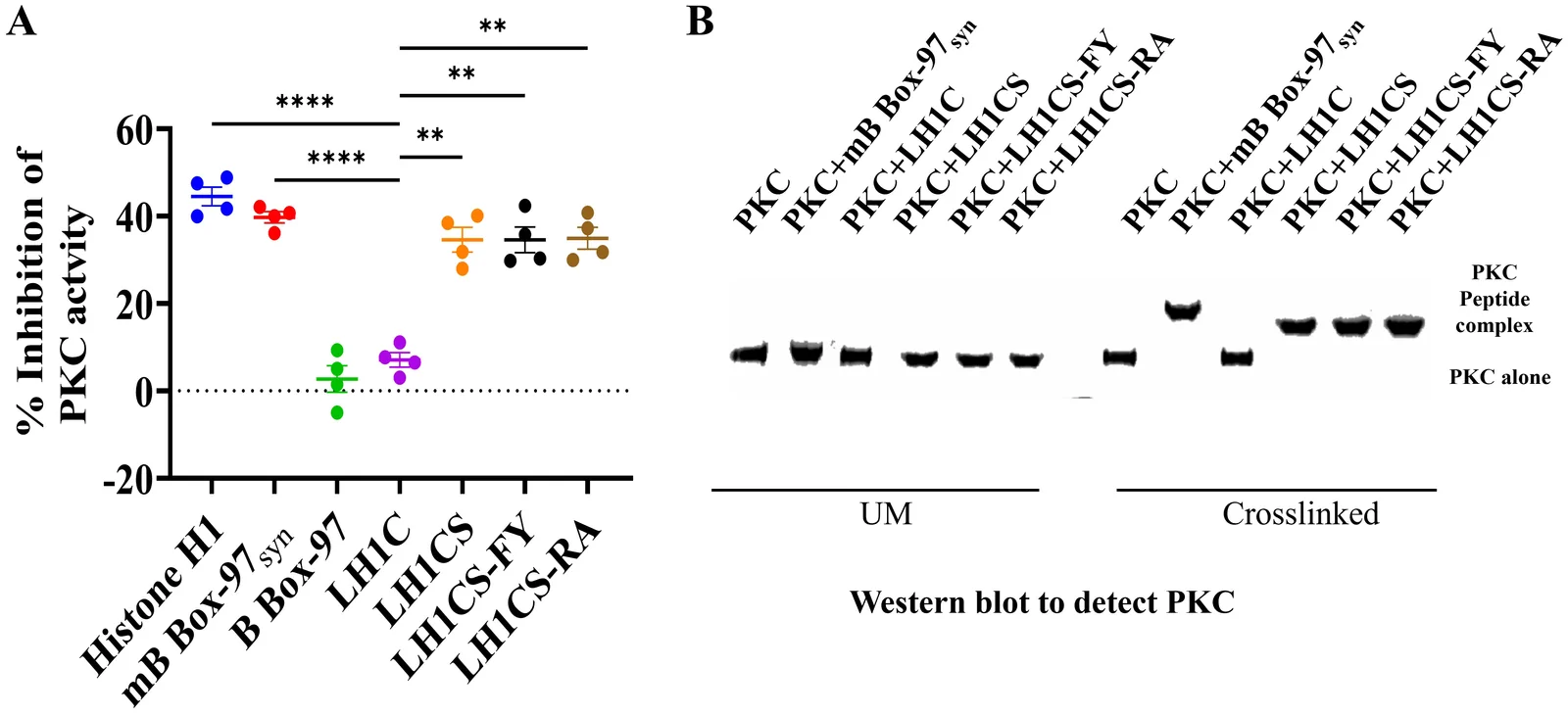
Interaction and inhibition of PKC by Helix-1 peptides. **(A)** PKC activity was assessed using a PKC activity assay kit in the presence of Histone H1/mB Box-97_syn_/B Box-97/LH1C/LH1CS/LH1CS-FY/LH1CS-RA. Histone H1 and mB Box-97 served as a positive control while B Box-97 is used as negative control. Mean values of n = 4 biological replicates ± SEM are shown. **(B)** Conditions unmodified (UM) and crosslinked with glutaraldehyde (crosslinked) are indicated. Post incubation with or without glutaraldehyde; PKC with or without peptides were separated using SDS-PAGE and transferred to nitrocellulose membrane and probed with anti-PKC antibodies to detect PKC. PKC crosslinked with mB Box-97_syn_ was used as positive control. Representative blot of 3 independent experiments is shown. P values (ns = P > 0.05, **P < 0.005, ****P < 0.0001) using Welch and Brown-Forsythe ANOVA followed by Dunnett’s T test.

We next examined the binding of PKC to Helix-1 peptides using glutaraldehyde crosslinking. Helix-1 peptides formed a high-molecular-weight (~80 kD) complex with PKC, similar to the complex formed with mB Box-97_syn_, which was used as a positive control (**Figure 4B**; **Supplementary Figure S7**). These findings are consistent with Helix-1 peptides binding PKC and inhibiting its kinase activity in a C106S-dependent manner, via the same PKC-inhibitory mechanism as the parent mB Box-97_syn_ peptide.

### 3.5 Helix-1 peptides modulate p47^phox^ phosphorylation and reduction in ROS production

Given the importance of ROS in PMA-induced NETosis, and the ability of Helix-1 peptides to inhibit PKC activity, we tested the effect of these peptides on ROS production by PMNs. Isolated PMNs were preincubated with Luminol, a chemiluminescent indicator of ROS (36). Cells were then activated using PMA alone or PMA in combination with B Box-97, mB Box-97_syn_, LH1C, or Helix-1 peptides; media alone served as a negative control. ROS production was measured every 5 minutes over a 2-hour period. Helix-1 peptides significantly inhibited PMA-induced ROS production in human PMNs (**Figure 5A**).

**Figure 5:**
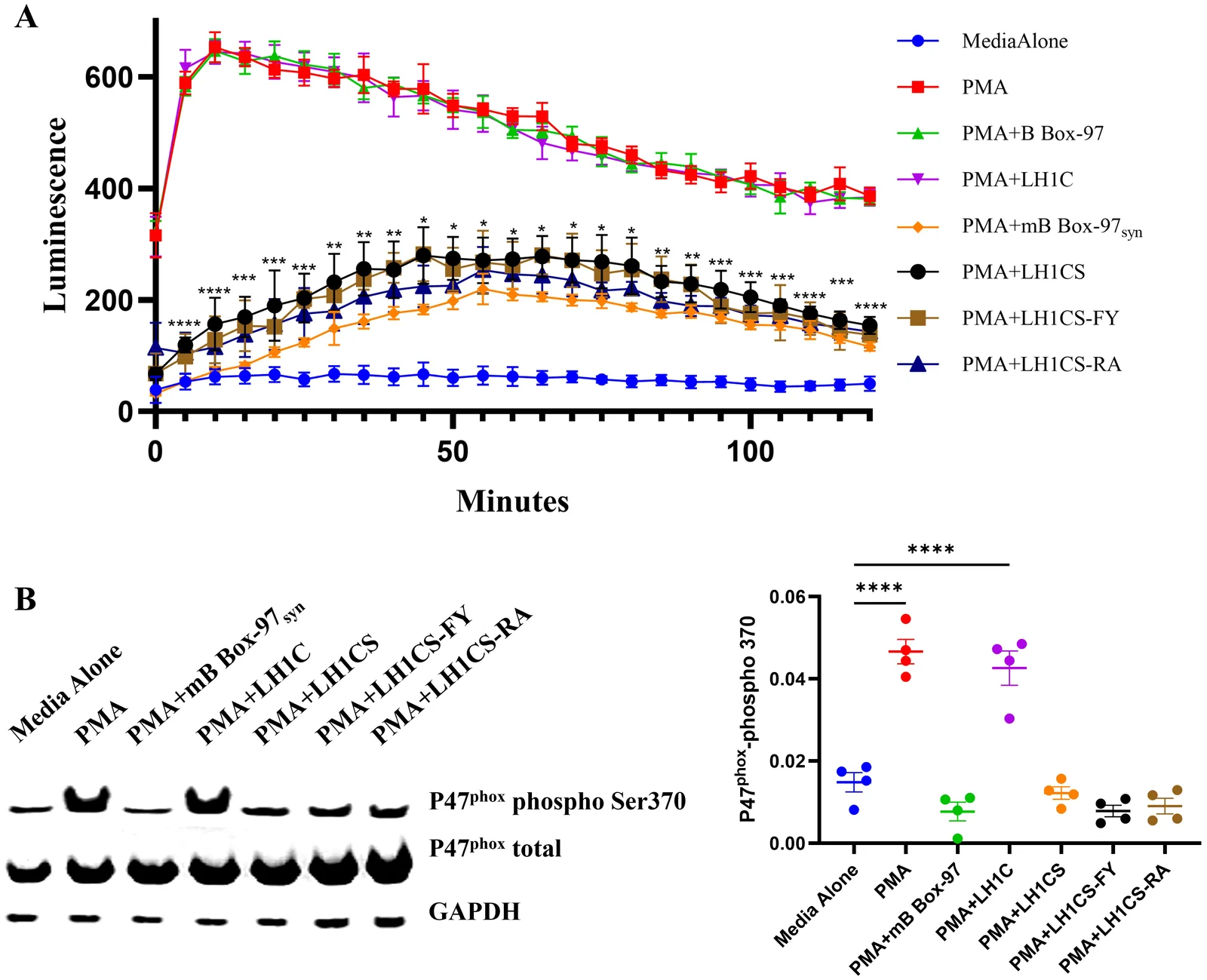
Helix-1 peptides inhibited P47^phox^ phosphorylation and ROS generation. **(A)** ROS as a measure of Luminol luminescence from PMNs in respective conditions was plotted against incubation time. mB Box-97_syn_ was used as positive control, LH1CS, LH1CS-FY and LH1CS-RA showed a significant reduction in ROS generation compared to PMA alone/PMA + B Box-97/PMA+LH1C treatment. Mean values of n = 4 biological replicates ± SEM are shown. **(B)** Quantification of steady state levels of phosphorylation of p47^phox^ on Ser-370 in PMNs post treatment under the described conditions was assessed using Western blotting. The intensity of each band was assessed using ImageJ and the relative intensity of phosphorylated p47^phox^ post normalization was plotted as Arbitrary Units (AU). Mean values of n = 4 biological replicates ± SEM are shown. (P values (NS >0.05 (not shown), *P<0.05, **P<0.05, ***P < 0.005, ****P < 0.0001) calculated using RM two-way ANOVA **(A)** or one-way ANOVA with Holm-Šídák’s multiple t-test **(B)**.

Phosphorylation of p47^phox^ is prerequisite for active assembly of NADPH oxidase and thereby production of ROS (10–12). To investigate the effect of Helix-1 peptides on p47^phox^ phosphorylation we treated human neutrophils with Helix-1 peptides, and extracted proteins were separated by SDS-PAGE and probed for phosphorylation of p47^phox^ at Ser370 using a phospho-Ser370-specific antibody by Western blotting. Total p47^phox^ and GAPDH were used as loading controls. Based on densitometric analysis, Helix-1 peptides significantly inhibited the phosphorylation of p47^phox^ while its wildtype counterpart LH1C peptide failed to show any similar effect (**Figure 5B**). Helix-1 peptides suppressed PMA-induced ROS production and reduced phosphorylation of the NADPH oxidase subunit p47^phox^, linking peptide-mediated PKC inhibition to downstream ROS suppression importantly to a similar extent as the parent mB Box-97_syn_ peptide.

### 3.6 Helix-1 peptides modulated bacterial killing by PMNs

Since Helix-1 peptides inhibited NETosis, PKC activity, ROS production, and release of the antimicrobial proteins NE and MPO, we tested whether Helix-1 peptides, similar to mB Box-97_syn_, also affect bacterial killing by PMNs (33). PMNs were incubated with buffer alone, or with Helix-1 peptides or LH1C, together with a 16-hour NTHI biofilm in which bacteria exist in equilibrium between a young biofilm state and a planktonic state due to natural biofilm remodeling as previously described (31). mB Box-97_syn_ and HU_NTHI_ served as positive controls. PMNs were lysed with Triton X-100 to recover viable intracellular bacteria, and total NTHI CFU was used to calculate the percent bacterial killing relative to the buffer-alone control. Because Triton X-100 lysis releases both phagocytosed bacteria and any bacteria retained within cell-associated NETs, this assay reports total PMN-associated killing rather than a NET-trap-specific readout. We have previously shown using this same biofilm-PMN killing assay that DNase I treatment, which dissolves NETs via degradation of eDNA tentacles released by NETs, significantly alters neutrophil-mediated bacterial killing (31) indicating this assay readout reflects killing attributable to PMN NETosis. Helix-1 peptides reduced PMN-mediated bacterial killing by 65% compared to LH1C, an effect comparable to that of HU_NTHI_ (~80% reduction) and mB Box-97_syn_ (~70% reduction) (**Figure 6**). Helix-1 peptides reduced PMN-mediated bacterial killing of NTHI biofilms to an extent comparable to mB Box-97_syn_ and the HU_NTHI_ positive control. This result indicates that Helix-1 peptide treatment impairs overall neutrophil-mediated antimicrobial function against NTHI biofilms, consistent with, though not exclusively diagnostic of, a downstream consequence of NETosis inhibition.

**Figure 6:**
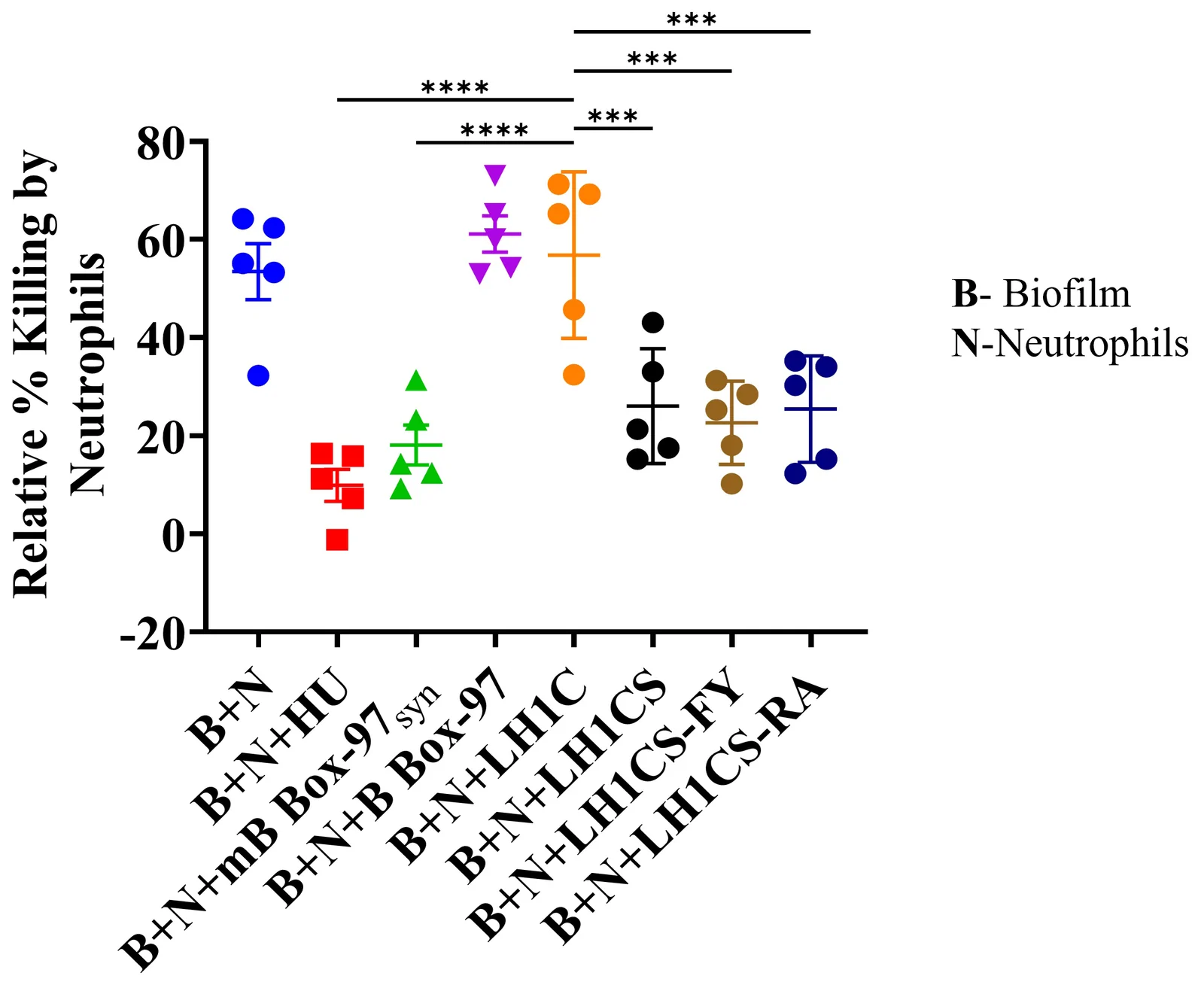
Neutrophil-mediated microbial killing is inactivated by Helix-1 peptides. NTHI biofilm was incubated with human neutrophils (B + N), or neutrophils treated with recombinant DNABII protein isolated from NTHI (B + N + HU_NTHI_) or mB Box-97_syn_ (B + N + mB Box-97_syn_) as a positive control, B Box-97 (B + N + B Box-97) served as a negative control. A control consisting of NTHI biofilm without neutrophil intervention was also included against which the relative percent killing was calculated. Higher values indicate greater bacterial killing (i.e., fewer viable bacteria recovered relative to no-neutrophil biofilm alone control); the negative controls (B Box-97, LH1C) and neutrophil-alone incubated with biofilm (B + N) therefore appear at the top of the graph because they do not impair PMN antibacterial function, whereas conditions that inhibit PMN function (positive controls and Helix-1 peptides) appear lower. LH1CS, LH1CS-FY and LH1CS-RA showed a significant inhibition of neutrophil mediated bacterial killing. Mean values of n = 5 biological replicates ± SEM are shown. P values (NS >0.05 (not shown), ***P < 0.005, ****P < 0.0001) calculated using one-way ANOVA with Holm-Šídák’s multiple T-test.

## 4 Discussion

We have previously characterized a 97 amino acid peptide derived from HMGB1 called mB Box-97_syn_. This peptide has the capacity to both prevent formation of and also disrupt existing bacterial biofilms through destabilizing the eDNA-dependent matrix structure and, in a completely different capacity, prevent NET formation. Both of these attributes have important clinical therapeutic value and here we determined that we could separate these two disparate functions into individual bioactive molecules.

The formation of NETs is an essential function of the innate immune system, and a growing body of literature has characterized both its protective role and the pathological consequences of its dysregulation (47). The balance between the beneficial induction of NETs and their timely clearance is critical to their protective effect. During NETosis, neutrophils release antimicrobial molecules that kill pathogens; however, because these molecules act nonspecifically, they can also injure surrounding tissue directly or by amplifying the inflammatory response. Indeed, excessive NET formation can worsen inflammation in autoimmune disease (4) and create a physical barrier to blood flow that contributes to atherosclerosis and stroke (19, 20, 48). In COVID-19, enhanced neutrophil infiltration, NET release, complement activation, and vascular thrombosis contribute to necroinflammatory lung injury, and elevated serum NETosis markers correlate with disease severity in mechanically ventilated patients (49, 50). Therapeutics that limit excessive NET formation may therefore reduce disease severity and improve survival across multiple conditions.

Likewise, pathogenic biofilms represent a severe pathologic state and are the primary cause of highly recalcitrant chronic and recurrent bacterial infections (51, 52). In the biofilm state, bacteria retreat into a community architecture typically containing multiple species that require an underlying eDNA matrix. The community architecture results in the resident bacteria becoming > 1000-fold resistant to antimicrobials than the free-living bacteria (27). Preventing formation of, or disrupting, the eDNA-dependent matrix releases the resident bacteria into a free and highly vulnerable state (53). We showed that the host molecule HMGB1 can prevent and disrupt bacterial biofilms by acting on the eDNA structure (26), while the host modulates HMGB1 release to merely cordon off biofilm proliferation instead of clearing them since HMGB1 is also an alarmin to the innate immune system and in excess will cause sepsis (54, 55). We were able to isolate the anti-biofilm activity without pro-inflammatory consequence in the same portion of HMGB1 as the anti-NETotic activity i.e. mB Box-97_syn_ (32, 33).

Our work here shows that these two disparate functions can indeed be separated and is a first step in understanding the minimum molecular requirements for each attribute. We previously showed that mB Box-97_syn,_ a 97 amino acid HMGB1-derived synthetic peptide carrying C106S mutation, retains the anti-biofilm activity of full length HMGB1 through its DNA-binding capacity, while its reduced proinflammatory activity is due in part to competitive inhibition of PKC by the engineered serine residue, which lowers ROS production and thereby inhibits NETosis (32, 33). In the present study, we showed that a minimized peptide corresponding to the linker region and helix 1 of mB Box-97_syn_ retains the parent peptides anti-NETotic activity, and that introducing DNA-binding mutations into this peptide separates its anti-NETotic activity from its anti-biofilm activity. All peptides used in this study were produced synthetically and are therefore free of post-translational modifications, an advantage further discussed below.

The DNA-binding and DNA-bending activity responsible for the anti-biofilm function of HMGB1 in part resides in its B Box domain (32, 34, 56), which is composed of three alpha helices. In our previous work, only full length, B Box-97-sized constructs that retained the linker sequence between the A Box and B Box domain showed significant biofilm disruption (32); the linker’s four positive charged lysine residues were proposed to facilitate DNA binding and consistent with this, the isolated A Box domain bends DNA more weakly than the B Box domain inclusive of the linker, which may promote electrostatic interactions in protein-DNA complexes (56, 57). In the bacterial biofilms, extracellular DNA (eDNA) serves as a key structural component, maintained in part by the bacterial DNABII proteins HU and IHF, which localize to the vertices of cross-stranded eDNA structures that functionally resemble Holliday junctions and are essential for biofilm structural integrity (43, 58). mB Box-97_syn_ binds these Holliday-junction-like structures to destabilize the eDNA dependent biofilm matrix (32).

In the present study, we constructed minimized helix peptides H1CS; H1,2CS; LH1CS; and LH1,2CS which retained the anti-biofilm activity of mB Box-97 regardless of whether the linker was present or not, which indicated that the linkers contribution to DNA binding in the full length mB Box-97 construct was not reproduced at the scale of this isolated helix. One possible explanation is that the anti-biofilm activity is, in part, limited by the last two alpha helices of the B-Box and that the linker region compensates for this limitation. Once the last two helices were removed, the linker region was dispensable; however, this remains to be tested directly. In contrast, the linker was indispensable for anti-NETotic activity; only peptides that carried both the linker and the C106S mutation inhibited NET formation, and this requirement is not explained by the DNA-binding model above. Because this linker also contains a cluster of positively charged residues and a known TLR-binding motif, we speculate that it may instead promote membrane association and cellular uptake of the peptide, but this remains untested and is an important direction for future work. We acknowledge that our conclusion that “the linker is essential for anti-NETotic activity” reflects a functional readout (NET inhibition) and does not itself distinguish a linker contribution to intracellular PKC engagement from a linker contribution to cellular/membrane uptake; because the linker’s positive charge is a hallmark of cell-penetrating peptides, differential uptake of LH1CS versus H1CS is a plausible alternative or additional explanation for their divergent anti-NETotic activity. A further consideration is that LH1C, which carries neither the R97A nor F103Y substitution used to disrupt DNA binding, also failed to bind HJ DNA and failed to disrupt biofilms. We have now directly confirmed the basis for this: LH1C forms a disulfide-linked dimer through its free, unmutated cysteine at position 106, and DTT-mediated reduction of this dimer to its monomeric form restored both HJ-DNA binding and anti-biofilm activity, establishing that disulfide-mediated dimerization, rather than a distinct structural defect in the DNA-binding surface, accounts for the LH1C phenotype. The linker’s precise mechanistic contribution to anti-NETotic activity, discussed above, remains an open question for future structural and biological study. Given the anti-biofilm activity via eDNA structure disruption appears to be retained by the helix one peptides, we further hypothesize that, like the parent mB Box-97, these constructs could also function additively or even synergistically with anti-DNABII antibodies that sequester the DNABII lynchpin protein, that serves to maintain the integrity of the eDNA structure, to more effectively clear biofilms; we plan to test these possibilities in future studies.

As there are disease conditions that involve excessive NETosis but lack a biofilm component, we reasoned that in these circumstances disrupting occult biofilms while inhibiting NETosis could be detrimental due to potential release of uncontrolled numbers of bacteria that could then overwhelm host immunity limited by inhibited PMN activity. Within the helix 1, R97 mediates DNA binding and F103 mediates intercalation into DNA (34, 35). The anti-biofilm activity of the peptide LH1CS relies on its DNA binding ability; therefore, to separate anti-NETotic activity from anti-biofilm activity, we individually mutated R97 and F103 to generate LH1CS-RA and LH1CS-FY, respectively. Both mutations abolished DNA binding and, consequently, anti-biofilm activity, while leaving anti-NETotic activity intact.

Reduced ROS generation by the helix-1 peptides is a significant finding because ROS drives the release of antimicrobial proteins from neutrophil granules, as well as the release of decondensed chromatin (59, 60). ROS triggers dissociation of neutrophil elastase (NE) from a membrane-associated complex into the cytosol and activates its proteolytic activity in an MPO-dependent manner (14). Activated NE then translocates to the nucleus, cleaving chromatin and releasing it into the cytoplasm to complete NET formation (1, 3). Because NET formation, antimicrobial release, and ROS generation together are responsible for microbial killing by NETs (3, 61, 62), the reduced release of NE and MPO we observed following peptide treatment is consistent with the reduced ROS generation caused by PKC inhibition.

PKC is a central regulator of NETosis, and its inhibition significantly impairs this process (1, 7, 63). PKC phosphorylates p47^phox^, promoting its translocation and assembly as part of the NADPH oxidase complex (12). We previously showed that the C106S mutation in mB Box-97_syn_, introduced to reduce proinflammatory activity, creates a potential new PKC phosphorylation site that allows mB Box-97_syn_ to act as a competitive PKC inhibitor perhaps by creating a futile cycle, reducing p47^phox^ phosphorylation, ROS generation, and NETosis (33). We hypothesized that the helix-1 peptides act through the same mechanism, and confirmed this: like mB Box-97_syn_, these peptides bound PKC and inhibited its activity, with corresponding reductions in p47^phox^ phosphorylation and ROS generation. Given that helix-1 peptides reduced both ROS generation and the release of NET-associated antimicrobial proteins, the accompanying reduction in neutrophil-mediated bacterial killing was an expected downstream consequence.

Few direct or indirect inhibitors of NET formation have reached preclinical testing, and none are currently FDA-approved for NET-related complications (50). Existing options include long-used clinical agents such as hydroxychloroquine, methotrexate, and prednisolone, as well as humanized antibodies including rituximab (anti-CD20), belimumab (a fully human IgG1λ recombinant antibody), and tocilizumab (anti-IL-6 receptor); other molecules inhibit NE, MPO, or PAD4 directly, though most function as indirect inhibitors of NETosis (50). Among PKC-targeted approaches, the PKC inhibitor ruboxistaurin (LY-333531) shows anti-NETotic activity against LPS-induced NETs in neutrophils from hospitalized COVID-19 patients (63), and Gö 6976 (a PKCα/β inhibitor), together with LY-333531 (a PKCβ-specific inhibitor), markedly reduces PMA-induced NET formation and ROS production in human neutrophils (7). Despite these advances, there remains a need for inhibitors of excessive NETosis that avoid off-target effects capable of impairing the beneficial functions of neutrophils.

Because Helix-1 peptides act upstream, at the level of PKC and NADPH oxidase activation, they are expected to blunt not only NETosis but also the ROS-dependent, phagocytic arm of neutrophil antimicrobial function that shares this same signaling node (10–13); the reduced PMN-mediated killing of NTHI biofilms that we observed is consistent with this broader effect. This raises an important safety consideration for any therapeutic strategy built on PKC-directed NETosis inhibition (4): because PKC and NOX2 activity support multiple, overlapping neutrophil effector functions, selectively inhibiting NETosis while fully preserving phagocytic and oxidative antimicrobial defenses may not be achievable with this mechanism, and systemic administration could in principle compromise host antibacterial immunity, particularly against biofilm-associated or other pathogens that rely on neutrophil ROS for clearance (11). We therefore envision Helix-1 peptides as most appropriate for localized, time-limited, or topical application in settings where excessive NETosis, rather than global antibacterial defense, is the dominant pathological driver (e.g., NET-mediated tissue injury in ischemic stroke (20) or severe COVID-19 (18)), and we consider a formal evaluation of systemic infection risk, using *in vivo* bacterial challenge models with monitored dosing and duration, a necessary prerequisite before broader therapeutic application; we discuss this further in the Limitations.

Together, our findings show that the linker and helix-1 region of mB Box-97 were jointly sufficient for anti-NETotic and anti-biofilm activity. Anti-NETotic activity could be separated from anti-biofilm activity by mutating key DNA-binding residues, and anti-biofilm activity alone could be localized to helix-1 without the linker. At only 42 and 29 residues, respectively, the resulting peptides can be readily synthesized at scale and are free of the post-translational modifications that could otherwise alter their activity or immunogenicity. These peptides represent the smallest HMGB1 derived peptides with specific anti-NETotic or anti-biofilm activity functions described to date. These findings further support PKC as a viable target for NETosis inhibition and warrant continued investigation. The ability to isolate anti-NETotic from anti-biofilm activity within a single, well characterized parent molecule provides a tractable, and to our knowledge novel, platform for developing functionally selective HMGB1 derived therapeutic for excessive NETosis, recalcitrant pathogenic biofilms or both.

## 5 Limitations

Several limitations temper these conclusions and should guide the next stage of this work.

First, all experiments were performed *in vitro* using neutrophils from a small pool of healthy donors. Whether these peptides retain their activity, selectivity, and lack of toxicity in neutrophils from patients with the diseases we invoke as potential indications (e.g. COVID-19, systemic lupus erythematosus, ANCA-associated vasculitis, ischemic stroke) remains untested, as does their behavior in any animal model of excessive NETosis or biofilm infection. To begin to address this, we plan a two-tiered follow-up approach: first, ex vivo testing of Helix-1 peptide activity (NETosis inhibition, PKC/ROS suppression, and biofilm disruption) in neutrophils isolated from patients with NET-associated conditions (e.g., ANCA-associated vasculitis or active COVID-19) and from patients with chronic NTHI/MRSA biofilm infections, benchmarked against matched healthy-donor neutrophils; second, *in vivo* evaluation in murine models of excessive NETosis (e.g., PMA- or ionophore-induced peritonitis, or a stroke/thrombosis model) and of chronic biofilm infection (e.g., NTHI otitis media), with peptide pharmacokinetics, dosing, and route of delivery established first in short-term tolerability studies.

Second, our anti-NETotic assays relied predominantly on PMA, a strong pharmacological PKC agonist. We did not systematically quantify peptide activity against more physiologically relevant, receptor-mediated NET triggers, such as LPS, immune complexes, or live pathogens, so the generality of PKC inhibition as a brake on NETosis across stimuli remains to be established. It is worth noting however, that the parent peptide mB Box-97 has shown inhibition of LPS and ionophore induced NETosis.

Third, the mechanistic model we propose that the DNA-binding residues R97 and F103 govern anti-biofilm activity independently of the C106S-dependent, PKC-mediated anti-NETotic mechanism does not fully account for our own data. The LH1C peptide, which carries neither DNA-binding mutations, unexpectedly lost both HJ-DNA binding and anti-biofilm activity. We have since confirmed that this is explained by disulfide-mediated dimerization of LH1C through its native, unmutated cysteine at position 106. DTT-mediated reduction restored both HJ-DNA binding and anti-biofilm activity (Section 3.2), establishing this off-target dimerization, rather than a defect in the proposed DNA-binding model, as the basis for the LH1C phenotype.

Fourth, our neutrophil-mediated killing assay (**Figure 6**) used Triton X-100 lysis to recover total PMN-associated bacteria, which does not distinguish bacteria killed within NETs from bacteria killed intracellularly following phagocytosis; consequently, the reduced killing we observed with Helix-1 peptides reflects impaired overall PMN antimicrobial function against the biofilm and cannot be just attributed to NETosis inhibition alone. As noted above, our prior finding that DNase I pretreatment alters neutrophil mediated killing in this assay (31) argues for a dominant NET-trapping contribution.

Fifth, we have not compared the peptides potency or selectivity against existing PKC-directed anti-NETotic agents such as ruboxistaurin or Gö 6976, leaving open the possibility of off-target effects on other PKC-dependent neutrophil or host functions. Finally, peptide stability was assessed but pharmacokinetics, immunogenicity, optimal route of delivery, and *in vivo* dosing are all unaddressed and will ultimately determine whether the favorable *in vitro* profile of these peptides translates into a viable therapeutic.

Addressing these gaps, particularly *in vivo* efficacy and safety testing, resolution of the linker’s mechanistic role, and head-to-head comparison with existing PKC-targeted therapeutics, will be necessary before the promise of these minimized peptides can be fully translated.

## Data Availability

The original contributions presented in the study are included in the article/**Supplementary Material**, further inquiries can be directed to the corresponding author/s.
