# Supplementary material for: Structure-based minimization of a high-mobility group protein B1 (HMGB1) B box peptide uncouples anti-NETotic activity and anti-biofilm function
Source: Front Immunol. 2026 Oct 16;17:1970789. doi: 10.3389/fimmu.2026.1970789 (PMC13719363; doi:10.3389/fimmu.2026.1970789)
Supplement: Supplementary file 1 [file SupplementaryFile1.docx]

**Supplementary Fig.1. Helix-1 peptides are stable in the Neutrophil media.** Representative silver-stained gel showing individual peptides post incubation for 4 hours at 37ᵒC with 10% human serum in HBSS buffer OR peptides in HBSS buffer OR conditioned media collected from the neutrophils which were induced to form NETs using PMA for 4 hours. The graphs on the left shows the quantification of degradation % compared to the loading control (peptide in HBSS buffer) of 4 independent experiments, N=4.

**Supplementary Fig.2. Short peptides derived from mB Box-97 disrupted NTHI biofilms.** 24-hour old NTHI biofilms were treated for 2 hours with or without respective constructs (200nM) and remaining biomass post treatment is shown. Data presented are mean ± SEM., N=3. Statistical significance compared to media alone control was assessed with one-way ANOVA using nonparametric Kruskal–Wallis test (with an uncorrected Dunn's test for multiple comparisons) (ns = P > 0.05 (not shown), *P < 0.05, **P < 0.01, and ***P < 0.001).

**Supplementary Fig.3. Effect of DTT on LH1C peptide dimerization and HJ-DNA binding.** (**A**)1µg LH1C/LH1CS peptide were incubated with 1mM DTT for respective time periods, separated using tricin gel and stained using silver stain. Molecular weights of markers are mentioned, and the position of dimer/monomers is shown. (**B**) Electrophoretic Mobility Shift Assay (EMSA) of LH1C and LH1CS peptide. 200nM peptides preincubated with or without 1mM DTT were incubated with HJ DNA are shown. Complexed HJ DNA with LH1C peptide treated with DTT demonstrated a slower relative migration pattern to free HJ DNA.

**Supplementary Fig.4. Effect of DTT on LH1C antibiofilm activity.** 24-hour old NTHI biofilms were treated for 2 hours with or without respective constructs (200nM) pretreated with DTT for 30 minutes and remaining biomass post treatment is shown. Data presented are mean ± SEM., N=3. Statistical significance compared to media alone control was assessed with one-way ANOVA using nonparametric Kruskal–Wallis test (with an uncorrected Dunn's test for multiple comparisons) (ns = P > 0.05 (not shown), **P < 0.01, and ***P < 0.001).

**Supplementary Fig.5. Effect of DTT on anti-NETotic activity of the small peptides.** (**A**) NETs were induced using PMA in the presence or absence of respective peptides (200nM) pretreated with 1mM DTT for 30 minutes and stained to detect DNA using SYTOX Green (green). NETs were visualized using CSLM at 20x magnification and were manually quantified using ImageJ, and the percentage of NET formation was calculated relative to total PMNs in each image area. Mean values of n = 3 biological replicates ± Standard error of the mean (SEM) is shown. (**B**) Fluorescence of DNA released from the PMNs induced with PMA with or without the addition of peptides (200nM) pretreated with 1mM DTT for 30 minutes was plotted as a percentage relative fluorescence compared to the untreated control group. Mean values of n = 3 biological replicates ± SEM are shown. P values (ns = P > 0.05 (not shown), *P < 0.05, **P < 0.01, ***P < 0.0005, ****P < 0.0001) are calculated using Welch and Brown-Forsythe ANOVA followed by Dunnett’s T-test.

**Supplementary Fig.6. Changes in Mean Fluorescence Intensity (MFI) of the NETs associated with proteins.** MFI values for NE and MPO were normalized with MFI for dsDNA and PM and plotted as a % of PMA alone treated control. Mean values of n = 3 biological replicates ± SEM are shown. P values (ns = P > 0.05, *p < 0.05, **p < 0.01) are from a Welch and Brown-Forsythe ANOVA followed by Dunnett’s T test.

**Supplementary Fig.7. Helix-1 peptides bind to PKC.** Conditions unmodified (UM) and crosslinked with glutaraldehyde (crosslinked) are indicated. Post incubation with or without glutaraldehyde; PKC with or without peptides were separated using SDS-PAGE and silver stained to detect various molecular complexes. PKC crosslinked with mB Box-97syn was used as positive control. The molecular weights of the markers, PKC peptide complexes, PKC alone and Peptide alone are shown. Representative gel of 3 independent experiments is shown.
